# IL-33 Drives Expansion of Type 2 Innate Lymphoid Cells and Regulatory T Cells and Protects Mice From Severe, Acute Colitis

**DOI:** 10.3389/fimmu.2021.669787

**Published:** 2021-07-15

**Authors:** Nhi Ngo Thi Phuong, Vittoria Palmieri, Alexandra Adamczyk, Robert Klopfleisch, Jost Langhorst, Wiebke Hansen, Astrid M. Westendorf, Eva Pastille

**Affiliations:** ^1^ Institute of Medical Microbiology, University Hospital Essen, University Duisburg-Essen, Essen, Germany; ^2^ Institute of Veterinary Pathology, Freie Universitaet Berlin, Berlin, Germany; ^3^ Department of Internal and Integrative Medicine, Klinikum Bamberg, Chair for Integrative Medicine, University of Duisburg-Essen, Bamberg, Germany

**Keywords:** colitis, intestinal inflammation, IL-33, ST2, Tregs, ILC2

## Abstract

The hallmarks of inflammatory bowel disease are mucosal damage and ulceration, which are known to be high-risk conditions for the development of colorectal cancer. Recently, interleukin (IL)-33 and its receptor ST2 have emerged as critical modulators in inflammatory disorders. Even though several studies highlight the IL-33/ST2 pathway as a key factor in colitis, a detailed mode of action remains elusive. Therefore, we investigated the role of IL-33 during intestinal inflammation and its potential as a novel therapeutic target in colitis. Interestingly, the expression of IL-33, but not its receptor ST2, was significantly increased in biopsies from the inflamed colon of IBD patients compared to non-inflamed colonic tissue. Accordingly, in a mouse model of Dextran Sulfate Sodium (DSS) induced colitis, the secretion of IL-33 significantly accelerated in the colon. Induction of DSS colitis in *ST2^-/-^* mice displayed an aggravated colon pathology, which suggested a favorable role of the IL 33/ST2 pathway during colitis. Indeed, injecting rmIL-33 into mice suffering from acute DSS colitis, strongly abrogated epithelial damage, pro-inflammatory cytokine secretion, and loss of barrier integrity, while it induced a strong increase of Th2 associated cytokines (IL-13/IL-5) in the colon. This effect was accompanied by the accumulation of regulatory T cells (Tregs) and type 2 innate lymphoid cells (ILC2s) in the colon. Depletion of Foxp3^+^ Tregs during IL-33 treatment in DSS colitis ameliorated the positive effect on the intestinal pathology. Finally, IL-33 expanded ILC2s, which were adoptively transferred to DSS treated mice, significantly reduced colonic inflammation compared to DSS control mice. In summary, our results emphasize that the IL-33/ST2 pathway plays a crucial protective role in colitis by modulating ILC2 and Treg numbers.

## Introduction

Inflammatory bowel disease (IBD) is a highly complex immune mediated disorder, which is characterized by uncontrolled chronic inflammation of the gastrointestinal tract (1). The prevalence of patients suffering from IBD is increasing remarkably over the last decades (2). Consequently, the identification of novel therapeutic strategies to prevent and treat this disease are of essential importance.

Interleukin (IL)-33, as a member of the IL-1 cytokine family, has emerged as a critical modulator in inflammatory disorders. IL-33 is constitutively expressed in the nuclei of different cell types such as endothelial, epithelial and fibroblast-like cells (3). Moreover, the high level expression in epithelial barrier tissues, such as the intestinal tract, suggests an important role for IL-33 during IBD (4). Upon tissue damage or pathogen encounter, IL-33 is released as an alarmin and signals through a heterodimer receptor that consists of serum STimulation-2 (ST2) (5) and IL-1 receptor accessory protein (IL-1RAcP). There are two splicing variants of ST2, one soluble form (sST2), which functions as a decoy receptor by binding directly to IL-33 to inhibit its signaling and the membrane-bound form (ST2L), by which IL-33 is able to activate the MyD88/NF-_K_B complex to modulate immune cell functions. *In vitro* and *in vivo*, IL-33 induces the production of the type 2 T helper cells (Th2)-associated cytokines IL-5 and IL-13 (6). ST2 is expressed on a variety of immune cells in the colon, including T and B cells, but also innate lymphoid cells, macrophages, dendritic cells and eosinophils (7). Hence, IL-33 release at mucosal barrier sites is able to activate different cells types, resulting in the induction of distinct immune response during inflammation. Therefore, understanding the interplay of IL-33 and ST2 in more detail might reveal new approaches to counteract intestinal inflammation (8).

Previous studies investigating the role of IL-33 during intestinal inflammation exhibited controversial results depending on the physiological context. On the one hand, IL-33 has been described to ameliorate intestinal inflammation in different mouse models of chemical induced colitis and to prevent *Clostridium difficile* associated colon pathology (9–12). On the other hand, deficiency of the IL-33/ST2 signalling pathway was shown to protect mice from DSS or TNBS induced colitis and exogenous IL-33 application was recognized to exacerbate bacterial induced colitis (13–15). Therefore, IL-33 is considered both host-protective as well as host-pathogenic in the gastrointestinal tract. The complex function of IL-33 in the intestine and the variety of different target cells seem to pose a challenge for the use of IL-33 as a therapeutic drug during colitis. Thus, defining the mechanisms of IL-33 signaling in the intestine still needs further research with specifically considering the interaction of ST2^+^ responding cells and their impact on intestinal inflammation.

In the present study, we used the murine DSS model to determine the function of IL-33 during acute colitis. Treatment with exogenous rmIL-33 tremendously ameliorated intestinal inflammation. Moreover, we identified that Tregs and ILC2s are the central target cells of IL-33 in the DSS-induced colitis model to drive tissue protection. In summary, our findings highlight the relevance of IL-33 during intestinal inflammation and support its potential as a therapeutic drug to restrain colitis.

## Materials and Methods

### Human Samples

Endoscopic mucosal biopsies were obtained from macroscopically inflamed or non-inflamed tissues of 13 IBD (3 MC and 10 UC) patients undergoing colonoscopy. Informed consent was obtained from all patients. Ethical approval was provided by the Faculty of the University of Duisburg-Essen (15-6183-BO).

### Mice

BALB/c mice were purchased from ENVIGO (Horst, Netherlands). *ST2^-/-^* and *RAG2^-/-^* mice were previously described (16, 17). DEREG/c mice express the diphtheria toxin (DT) receptor and the GFP exclusively under the control of the Fopx3 promoter (18). All transgenic mice were on BALB/c background, and bred and maintained under specific pathogen-free conditions at the local Animal Facility of University Hospital Essen. Mice were either co-housed (for females), or soiled bedding was exchanged every 2 – 3 days (for males), 2 weeks prior to and during experiments. All animal experiments performed in this study were carried out in accordance with the ethical principles and guidelines for scientific experiments and were approved by the local Landesamt fuer Natur-, Umwelt- und Verbraucherschutz (LANUV, North-Rhine-Westphalia, Germany).

### Induction of Intestinal Inflammation

To induce intestinal inflammation, mice received 3 – 4% DSS (MP Biomedicals, Heidelberg, Germany, MW, 36-50 kDa) orally *via* the drinking water for 6 days followed by one day with normal drinking water. Mice were weighed and monitored daily for signs of sickness. The disease activity index (DAI, 0-12) was determined as the combined score of weight loss compared to initial weight, stool consistency, and rectal bleeding. Scores were defined as follows: weight loss: 0 (no loss), 1 (1-5%), 2 (5-10%), 3 (10-20%), and 4 (>20%); stool consistency: 0 (normal), 2 (loose stool), and 4 (diarrhea); and bleeding: 0 (no blood), 2 (visual pellet bleeding), and 4 (gross bleeding, blood around anus) (19).

### *In Vivo* Treatments

Mice were treated i.p. with recombinant mIL-33 (1 µg/mouse, BioLegend, San Diego, USA) diluted in 200 µl PBS on day 0, 2 and 5 of DSS application. In DEREG/c mice, the IL-33 treatment was performed on day 1, 3 and 6. For the depletion of Foxp3^+^ Tregs, DEREG/c mice were injected i.p. with diphtheria toxin (Merck, Darmstadt, Germany, 750 ng per mouse) on day 0, 2 and 5 of DSS application. To investigate the impact of ILC2 cells, BALB/c WT mice were treated i.p. with 1 µg recombinant mIL-33 on day 0, 2, 4 and 6 to expand ILC2s *in vivo*. Sorted ILC2s from splenocytes (2 x 10^5^ per mouse) were injected i.v. on day 0 in BALB/c mice, simultaneously to the DSS administration.

### Isolation of Immune Cells From Colonic Lamina Propria

Lamina propria lymphocytes (LPLs) were isolated from the colon. Therefore, colons were flushed with PBS to remove feces, opened longitudinally and cut into 1 cm pieces. Tissue pieces were washed twice in PBS containing 3 mmol/l EDTA rotating for 10 min at 37°C. To remove the EDTA, tissue pieces were further washed twice in RPMI-1640 containing 1% FCS, 1 mM EGTA and 1.5 mmol/l MgCl_2_ rotating for 15 min at 37°C. Subsequently, colon pieces were rinsed with PBS, minced and digested with RPMI-1640 containing 20% FCS and 100 U/ml collagenase IV (Merck) rotating for 60 min at 37°C. After filtration through a 40-µm filter to separate single cells from the remaining tissue, cells were washed with RPMI-1640 and finally suspended in culture medium for further analysis.

### Antibodies and Flow Cytometry

Flow cytometry analysis of surface proteins was conducted by using fluorochrome-labeled antibodies against CD11b (M1/70), CD11c (HL3), CD19 (1D3), CD4 (RM4-5), CD11b (M1/70), SiglecF (E50-2440), Gr-1 (RB6-8C5) (all BD Biosciences, Heidelberg, Germany) and CD3 (145-2C11), CD49b (DX5), ICOS (C398.4A), CD45 (30-F11) (all BioLegend) and ST2 (RMST2-2) (both Thermo Fisher Scientific, Braunschweig, Germany). Lineage^-^ antibody-cocktail was used for the ILC2 staining and contained antibodies against CD3, CD19, CD11c, CD49b, Gr-1 and CD11b. Viable and dead cells were separated using the Fixable Viability Dye eFluor 780 (Thermo Fisher Scientific). Intracellular staining was performed using the Foxp3 staining kit (Thermo Fisher Scientific), anti-Foxp3 (FJK-16s) and anti-GATA-3 (TWAJ) (both Thermo Fisher Scientific) according to the manufacturer’s recommendation. All flow cytometry analyses were performed on a LSR II instrument using the DIVA software (BD Biosciences).

### RNA Extraction and Quantitative RT-PCR

RNA was isolated from colon biopsies using the RNAeasy Fibrous Tissue Kit (Qiagen, Hilden, Germany) according to the manufacturer’s recommendations. To synthesize cDNA, 1 µg of RNA was reversed transcribed using M-MLV Reverse Transcriptase (Promega, Mannheim, Germany) with dNTPs, Oligo-dT mixed with Random Hexamer primers (Thermo Fisher Scientific). Quantitative RT-PCR was conducted using the SYBR Green PCR Kit (Thermo Fisher Scientific) and specific primers for mice including mouse *Il33* (5’-CTA CTG CAT GAGA CTCC GTT CTG-3’ and 3’-AGA ATC CCG TGG ATA GGCA GAG-5’), *sSt2* (5’-GTG GGT CGTC TGC AGA AAT-3’ and 3’-GCT CTC TGA GGT AGGG TCCA-5’), *St2l* (5’-TGT GAG CCG TGT GAG TTT GAG TGT-3’ and 3’ TGG AGC AGC AGG CAT GAGG AAGC-5’), *Retnlb* (5’-GAA CGC GCA ATG CTC CTTT GAG-3’ and 3’-AGC CAC AAG CAC ATC CAGT GAC-5’), *Spdef* (5’-CAC GTTG GAT GAG CAC TCG CTA-3’ and 3’-AGC CAC TTC TGC ACG TTAC CAG-5’), *Muc2* (5’GCT GAC GAG TGG TTG GTGA ATG-3’ and 3’-GAT GAG GTGG CAG ACA GGA GAC-5’) and for human samples *Il33* (5’-GGA AGA ACA CAG CAA GCA AAG CCT-3’ and 3’-TAA GGC CAG AGC GGA GCT TCA TAA-5’), *sSt2* (5’-GAA AAA ACG CAA ACCT AACT-3’ and 3’-TCA GAA ACA CTCC TTA CTTG-5’) and *St2l* (5’-AGG CTT TTC TCT GTT TCC AGT AAT CGG-3’ and 3’-GGC CTC AAT CCA GAA CAT TTTT AGG ATGA TAAC-5’). Relative mRNA levels were defined by using included standard curves for each individual gene and further normalization to the housekeeping gene ribosomal protein S9 (*Rps9*, mouse 5’-CTG GAC GAG GGC AAG ATGA AGC-3’ and 3’-TGA CGT TGG CGG ATG AGC ACA-5’, human 5’-CGC AGG CGC AGA CGG TGGA AGC-3’ and 3’-CGA AGG GTC TCC GCG GGG TCA CAT5-’).

### Cytokine Measurement in Colonic Explants

To quantify cytokine levels, colonic biopsies were cultured *in vitro* for 6 hours in 300 µl of IMDM medium supplemented with 10% FCS, 100 µg/ml Streptavidin, 100 U/ml Penicillin, 25 µM β-Mercaptoethanol. Secreted proteins in the supernatants were quantified by polystyrene bead-based Luminex technology (R&D Systems, Minneapolis, USA) according to the manufacturer’s instructions. The concentrations were calculated using the Luminex 200 instrument and the Luminex xPOTENT software (Luminex Corporation, Austin, USA) and normalized to the respective colon biopsy weight.

### Histopathological Analysis

Full-length colons were rinsed in ice-cold PBS and fixed in 4% paraformaldehyde, followed by embedding in paraffin and sectioning at 4 µm thickness. After staining with hematoxylin and eosin (H&E) the severity of the histopathology was scored in a blinded manner according to the inflammation markers, including inflammatory cell infiltration, epithelial defects, goblet cell depletion, granulocyte infiltration, crypt abscess, hyperplasia and ulceration. The colons were divided into oral, middle and rectal part and each marker was scored from 0 (= no signs of inflammation) to 3 (= severe signs of inflammation).

### Statistical Analysis

Normality of results was tested using D’Agostino and Pearson and Shapiro-Wilk normality test. Statistical analysis was calculated using Student’s t-test, Mann-Whitney test, Wilcoxon matched-pairs signed rank test, one-way ANOVA or two-way ANOVA, followed by Tukey’s, Dunn’s, Dunnett’s multiple comparison test or Sidak post-test. All analyses were performed using the Prism 7.03 software (Graphpad, La Jolla, CA). Statistical significance were indicated with *P ˂ 0.05, **P < 0.01 and ***P < 0.001.

## Results

### IL-33 Is Elevated in the Colonic Tissue of IBD Patients and in Mice With Acute Colitis

**Figure 1:**
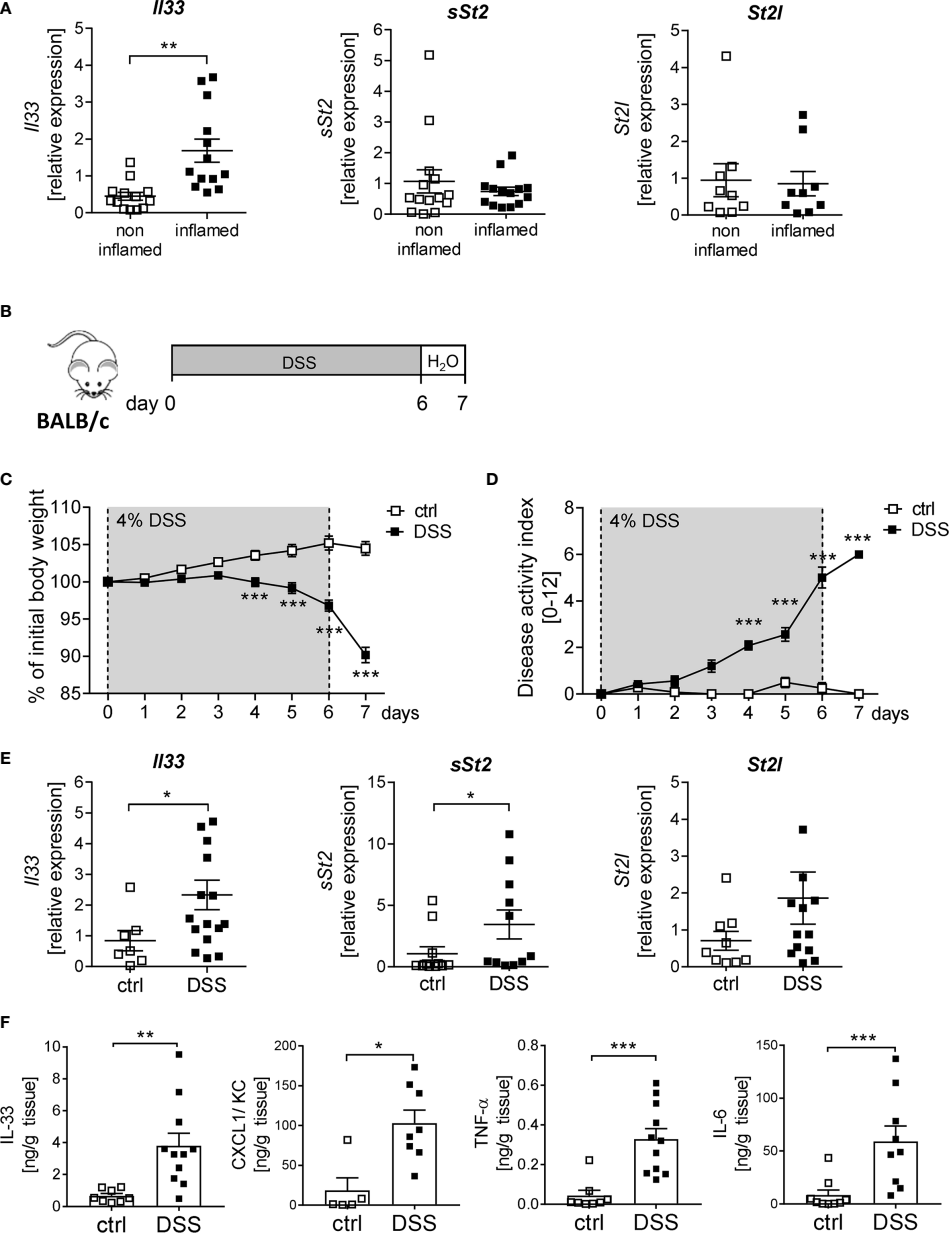
Elevated levels of IL-33 in IBD patients and mice with acute colitis. **(A)** Colonic mRNA expression of *Il33*, soluble *ST2 (sST2*) and membrane-bound *St2 (ST2l)* were measured by quantitative RT-PCR in non-inflamed and inflamed tissue of IBD patients (n = 9-14). **(B)** To induce intestinal inflammation BALB/c mice were administered 4% of DSS *via the* drinking water for 6 days, followed by one day of normal drinking water. **(C)** Body weight loss and **(D)** disease activity index were monitored daily. **(E)** Colonic mRNA expression of *Il33, sST2* and *ST2l* of healthy BALB/c mice (ctrl) and mice with DSS-induced colitis were measured by quantitative RT-PCR on day 7 (n = 7-15 mice per group. **(F)** Colonic explants were cultured for 6h and secreted cytokines (IL-33, CXCL1/KC, TNF-α and IL-6) were measured in the supernatants using Luminex technology. All data are presented as mean ± SEM. Statistical analyses were performed using Wilcoxon matched-pairs signed rank test **(A)**, two-way ANOVA followed by Sidak post-test **(C, D)**, Mann-Whitney test (**E**, **F** for CXCL1, TNF-α and IL-6), or unpaired Student’s t-test (**F** for IL-33). *P < 0.05; **P < 0.01; ***P < 0.001.

IL-33 is described to be important for immune cell activation upon tissue injury at mucosal surfaces (8). To determine the function of IL-33 in IBD, we first analyzed the expression of IL-33 and its receptor ST2 in human colonic biopsies. Interestingly, we observed a significant upregulation of *Il33* expression in inflamed tissues compared to non-inflamed control tissue. In contrast, no alterations in the expression levels of the soluble form of *St2* (*sSt2*) or the membrane-bound isoform of *St2* (*St2l*) was detected (**Figure 1A**). To address the functional role of IL-33 in more detail, a murine DSS-induced colitis model was used. BALB/c mice were orally administered with 4% of DSS in the drinking water for 6 days, followed by one day of normal drinking water (**Figure 1B**). At day 4-7, DSS treated mice developed severe inflammation indicated by a strong body weight loss and a high disease activity index (**Figures 1C, D**). Consistent with the data obtained from IBD patients, colonic *Il33* expression was significantly upregulated on mRNA as well as on protein levels in DSS treated mice compared to healthy control mice (**Figures 1E, F**). As expected for the DSS model, pro-inflammatory chemokines and cytokines, like CXCL1/KC, TNF-α and IL-6, were strongly induced due to DSS-treatment (**Figure 1F**). Of note, in contrast to IBD patients, we observed a slight increase in *sSt2* and *St2l* expression in the colon of DSS mice (**Figure 1E**).

### ST2 Protects Mice From Intestinal Inflammation During DSS-Induced Colitis

**Figure 2:**
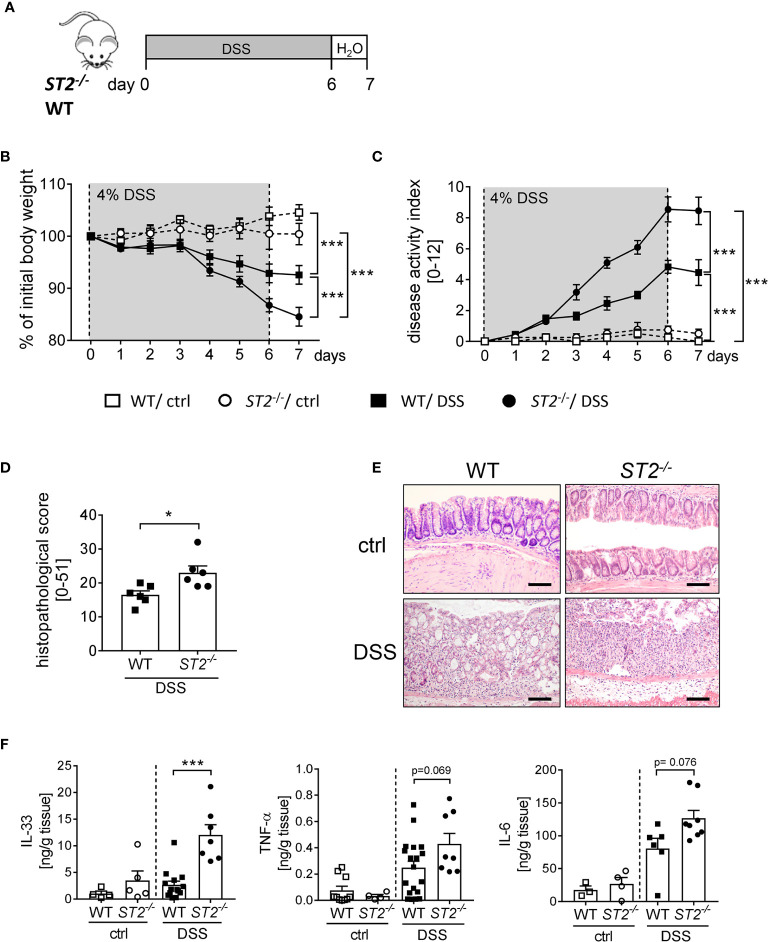
ST2 deficiency amplified the susceptibility to DSS-induced colitis. **(A)** DSS was orally administered to *ST2^-/-^* mice and BALB/c WT mice (n = 10-14 mice per group) for 6 days, followed by one day of normal drinking water. **(B)** Body weight changes relative to the initial weight and **(C)** disease activity index were monitored daily. **(D)** Histopathological score (n = 6) and **(E)** representative Hematoxylin and Eosin (H&E)-staining pictures of healthy control (ctrl) and inflamed colons (DSS) were evaluated on day 7. Scale bars represent 100 µm. **(F)** Secreted levels of IL-33, TNF-α and IL-6 were determined in the supernatants of colonic explants *via* Luminex technology. All data are presented as mean ± SEM. Statistical analyses were performed using two-way ANOVA, followed by Tukey’s multiple comparison test **(B, C)**, unpaired Student’s t-test **(D)**, or one-way ANOVA followed by Dunn’s or Dunnett’s multiple comparison test **(F)**. *P < 0.05; ***P < 0.001.

To further elucidate the role of IL-33 during intestinal inflammation, we applied the DSS-induced colitis model to ST2 deficient (*ST2* ^-/-^) mice (**Figure 2A**). Interestingly, *ST2^-/-^* mice suffered more from acute colitis compared to wildtype (WT) mice as demonstrated by significant higher body weight loss (**Figure 2B**), higher disease activity indices at day 7 (**Figure 2C**) and increased histopathological scores (**Figures 2D, E**) compared to DSS treated WT mice. Well in line, DSS-treated mice deficient for ST2 showed an amplified pro-inflammatory cytokine profile in comparison to WT mice, as we found significant stronger secretion of IL-33 and IL-6 and in tendencies more TNF-α in the supernatant of colonic explants of DSS treated *ST2^-/-^* mice (**Figure 2F**). Taken together, the absence of the IL-33/ST2 pathway augmented disease severity, suggesting a host-protective role for IL-33 during DSS-induced colitis.

### Exogenous IL-33 Application Orchestrates an Immune Network to Counteract DSS-Induced Intestinal Inflammation

**Figure 3:**
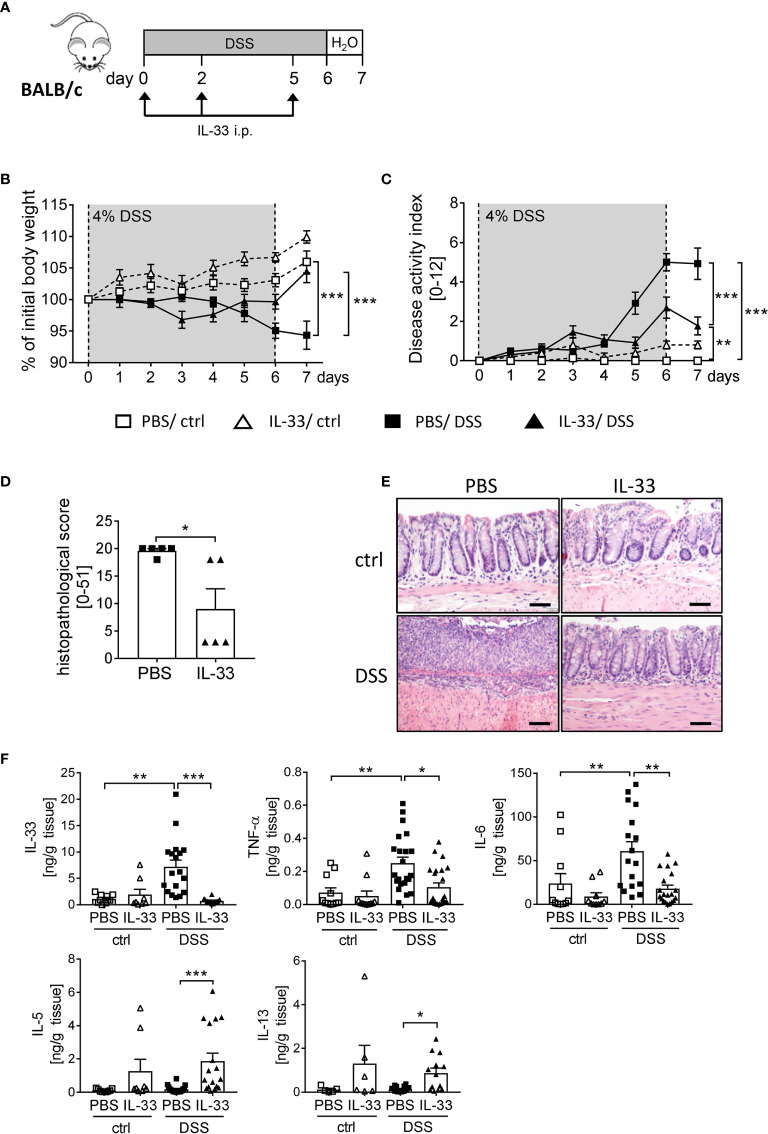
IL-33 administration alleviates intestinal inflammation. To induce intestinal inflammation, BALB/c mice (n = 7-13 mice per group) were given DSS *via* the drinking water for 6 days, followed by one day of normal drinking water. **(A)** On day 0, 2 and 5 control mice as well as DSS mice were i.p. injected either with recombinant mIL-33 or with PBS. **(B)** Body weight loss and **(C)** disease activity index were monitored daily. **(D)** Histopathological scores of PBS and IL-33-treated DSS-mice were determined on day 7 (n = 5 – 13 mice per group). **(E)** Representative Hematoxylin and Eosin (H&E) stained colon sections at day 7. Scale bars represent 100 µm. **(F)** Cytokine levels of IL-33, TNF-α, IL-6, IL-5 and IL-13 were measured in the supernatants of colonic explants by Luminex technology. All data are presented as mean ± SEM. Statistical analyses were performed using two-way ANOVA followed by Tukey’s multiple comparison test **(B, C)**, one-way ANOVA followed by Dunn’s multiple comparison test **(F)**, or using Mann-Whitney test **(D)**. *P < 0.05; **P < 0.01; ***P < 0.001.

To boost the IL-33/ST2 signaling pathway, we injected BALB/c WT mice with recombinant murine IL-33 during DSS treatment (**Figure 3A**). IL-33-treated healthy control mice showed no signs of intestinal inflammation (**Figures 3B, C**). Importantly, IL-33 treatment had a remarkable impact during DSS-induced colitis. Mice treated with IL-33 showed a significant improved body weight at day 6 compared to DSS control mice (PBS/DSS), which was even more prominent at day 7 when disease pathology was most severe (**Figure 3B**). PBS/DSS mice suffered from severe diarrhea, rectal bleeding and body weight loss, which is summarized in the disease activity index. IL-33-treated DSS (IL-33/DSS) mice showed a significant lower disease activity index already from day 5 on, when PBS/DSS mice started to show signs of sickness (**Figure 3C**). Additionally, the histopathological score was significantly reduced upon IL-33 treatment in comparison to PBS/DSS mice, indicated by less inflammatory cell infiltration, less epithelial defects and a lower degree of ulceration (**Figures 3D, E**). Accordingly, IL-33/DSS mice secreted lower levels of pro-inflammatory colonic cytokines such as IL-33, TNF-α and IL-6. In contrast, levels of the Th2 associated cytokines IL-5 and IL-13 were strongly enhanced upon IL-33 treatment, irrespective of DSS administration (**Figure 3F**). Collectively, these findings demonstrated that treatment with recombinant IL-33 is able to protect mice from severe intestinal pathology induced by DSS.

### The Protective Effect of IL-33 on DSS-Induced Colitis Is Partially Dependent on Tregs

**Figure 4:**
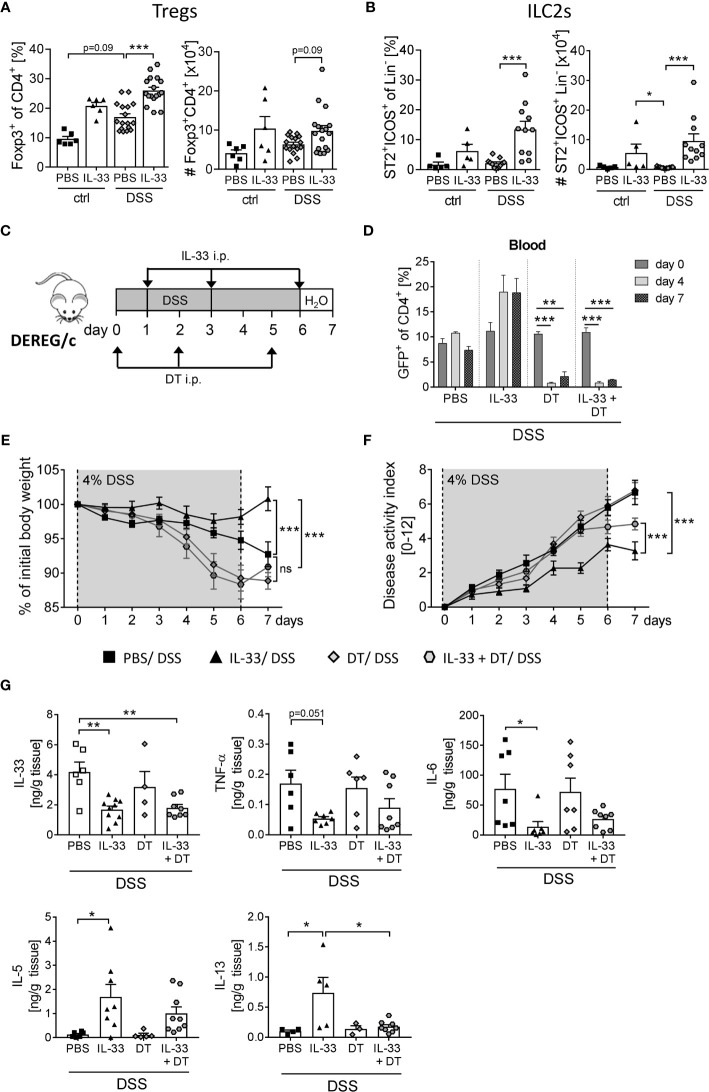
Treg depletion diminishes the protective effect of IL-33 in DSS colitis. To induce intestinal inflammation, BALB/c mice (n = 5-14 mice per group) were given DSS *via* the drinking water for 6 days. Followed by one day of normal drinking water. On day 0, 2 and 5 control mice as well as DSS mice were injected i.p. either with recombinant mIL-33 or with PBS. Immune cells were isolated from the colonic lamina propria on day 7 and analyzed by flow cytometry to distinguish **(A)** CD4^+^Foxp3^+^ regulatory T cells (Tregs), or **(B)** lineage^-^ICOS^+^ST2^+^ type 2 innate lymphoid cells (ILC2s). Frequencies and absolute numbers of cells were determined. **(C)** To induce intestinal inflammation in DEREG/c mice, DSS was given for 6 days, followed by one day of normal drinking water (n = 9-12 mice per group). Tregs were depleted in DEREG/c mice by injecting DT i.p. on day 0, 2 and 5. Mice were additionally treated either with PBS or with recombinant murine IL-33 i.p. on day 1, 3 and 6. **(D)** Treg ablation was confirmed in the blood on day 0, 4 and 7. **(E)** Change of body weight and **(F)** disease activity index were monitored daily. **(G)** Secretion of IL-33, TNF-α, IL-6, IL-5 and IL-13 in colonic explants was detected by Luminex technology. All data are presented as mean ± SEM. Statistical analyses were performed using one-way ANOVA followed by Dunn’s multiple comparison test **(A, B)**, two-way ANOVA followed by Tukey’s multiple comparison test **(D–F)**, or using one-way ANOVA followed by Tukey’s or Dunn’s multiple comparison test **(G)**. *P < 0.05; **P < 0.01; ***P < 0.001.

ST2, the receptor for IL-33, is expressed on a variety of immune cells in the colon (7). By gating CD4^+^ Foxp3^+^ cells (**Supplementary Figure 1A**) we observed a moderate increase in frequencies and absolute number of Tregs in the colon upon DSS-induced colitis (**Figure 4A**). Interestingly, colonic ST2^+^ ICOS^+^ ILC2 cells were not affected by DSS-induced inflammation (**Figure 4B**). However, the application of IL-33 significantly enhanced frequencies and absolute numbers of colonic Tregs and ST2^+^ ICOS^+^ ILC2s in DSS mice (**Figures 4A, B**). As it is known that ILC2 surface marker expression is heterogeneous and context-dependent (20), we verified a stable ILC2 phenotype by intracellular staining for the transcription factor GATA-3 (**Supplementary Figure 1B**).

To first evaluate the role of IL-33-mediated Treg expansion on the course of DSS-induced colitis, we used DEREG/c mice to specifically deplete Foxp3^+^ Tregs by applying diphtheria toxin (DT) (18). DEREG/c mice were treated with 4% of DSS in the drinking water for 6 days to induce acute colitis. Concomitantly, mice were injected with DT at day 0, 2 and 5 with or without recombinant IL-33 at day 1, 3 and 6 (**Figure 4C**), and depletion of Tregs after DT injection was confirmed in the blood. Independent of IL-33 administration, DT application efficiently eliminated Tregs from day 4 onwards (**Figure 4D**). Treg-depleted mice developed severe intestinal inflammation, which was slightly but not significant stronger when compared with PBS-treated DSS mice (**Figures 4E, F**). Interestingly, treatment with IL-33 in Treg-depleted DSS mice showed no improve in disease outcome in terms of body weight, but slightly reduced disease activity index compared to Treg-depleted DSS mice that did not receive IL-33 (**Figure 4F**). Nevertheless, alleviation of intestinal inflammation was not as prominent as in IL-33-treated Treg-sufficient mice. Well in line, IL-33 treatment resulted in a reduction of IL-33, TNF-α and IL-6 production in Treg-sufficient mice, which could also be detected in Treg-depleted mice. Conversely, upregulation of IL-33-induced Th2 cytokines IL-5 and IL-13 was abrogated by Treg depletion (**Figure 4G**). Taken together, these data suggest that the protective effect of IL-33 during colitis could be, at least in part, dependent on Tregs.

### IL-33 Acts on Innate Immune Cells to Protect Mice From DSS-Induced Intestinal Inflammation

**Figure 5:**
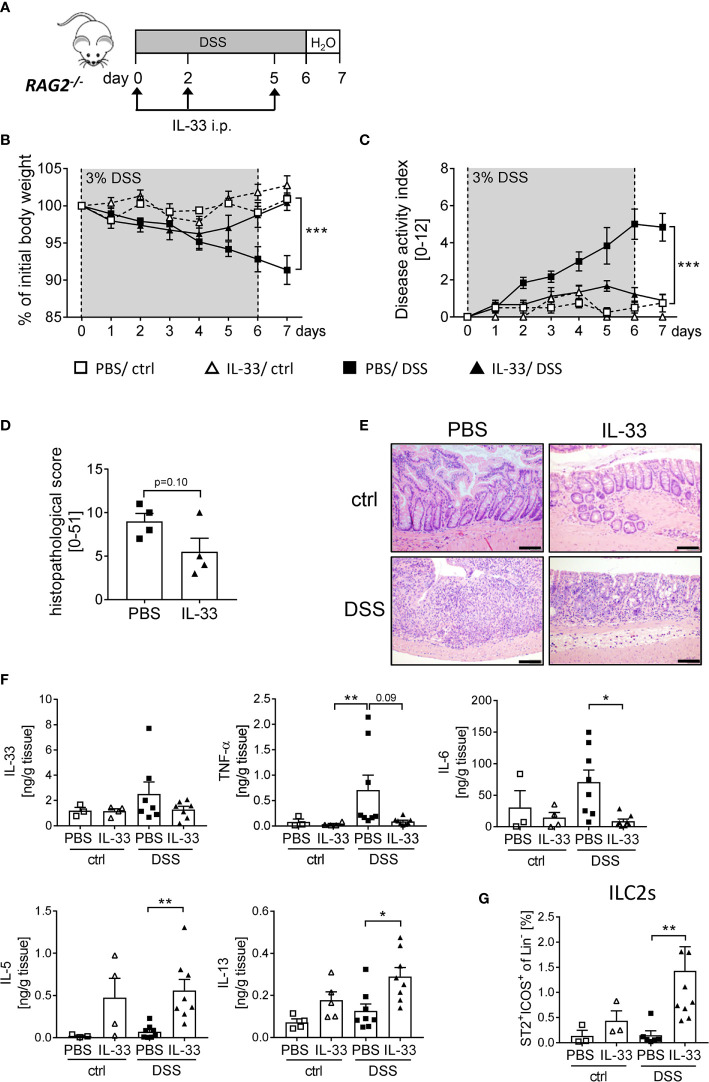
Innate immune cells protect mice from DSS-induced colitis upon IL-33 treatment. **(A)** To investigate the role of innate immune cells, DSS was administered to *RAG2^-/-^* mice for 6 days, followed by one day of normal drinking water. Mice were injected i.p. either with PBS or with IL-33 on day 0, 2 and 5 (n = 3-9 mice per group). **(B)** Body weight change and **(C)** disease activity index were monitored daily. **(D)** Histopathological score of PBS and IL-33-treated *RAG2^-/-^* mice was assessed on day 7, and **(E)** representative H&E staining of colon sections were analyzed (n = 4). Scale bars represent 100 µm. **(F)** Levels of IL-33, TNF-α, IL-6, IL-5 and IL-13 were measured in colonic explants using Luminex technology. **(G)** Immune cells were isolated from the colonic lamina propria and the frequencies of lineage^-^ICOS^+^ST2^+^ ILC2s were analyzed by flow cytometry. All data are presented as mean ± SEM. Statistical analyses were performed using two-way ANOVA followed by Tukey’s multiple comparison **(B, C)**, unpaired Student’s t-test **(D)**, or one-way ANOVA followed by Dunn’s or Dunnett’s multiple comparison test **(F, G)**. *P < 0.05; **P < 0.01; ***P < 0.001.

As the protective effect of IL-33 treatment was only partially dependent on Tregs, we further assessed the overall importance of adaptive immune cells using *RAG2^-/-^* mice, which lack mature T and B cells. We induced acute colitis by orally administering 3% of DSS for 6 days and treated *RAG2^-/-^* mice with recombinant IL-33 on day 0, 2 and 5 (**Figure 5A**). Surprisingly, we observed a similar disease progression as in WT mice. *RAG2^-/-^* mice developed significantly less DSS-induced inflammation upon IL-33 treatment compared to control mice (**Figures 5B, C**). In accordance, the histopathological score was ameliorated in IL-33 treated DSS mice in comparison to PBS-treated DSS mice (**Figures 5D, E**) with diminished pro-inflammatory cytokine production (**Figure 5F**). Although *RAG2^-/-^* are deficient in T cells, IL-33 treatment was sufficient to reduce the magnitude of DSS-induced intestinal inflammation. These results demonstrate that besides CD4^+^ Foxp3^+^ Tregs, other innate immune cells must be involved in the protective effect of IL-33. Importantly, IL-33 treatment promoted colonic IL-5 and IL-13 release, as well as the expansion of ILC2s in ctrl or DSS treated *RAG2^-/-^* mice (**Figures 5F, G**). Thus, we also focused on the function of ILC2s as key regulators in counteracting DSS-induced colitis upon IL-33 treatment.

### ILC2s Promote a Protective Phenotype Against Intestinal Inflammation Through the Induction of Intestinal Goblet Cell Differentiation

**Figure 6:**
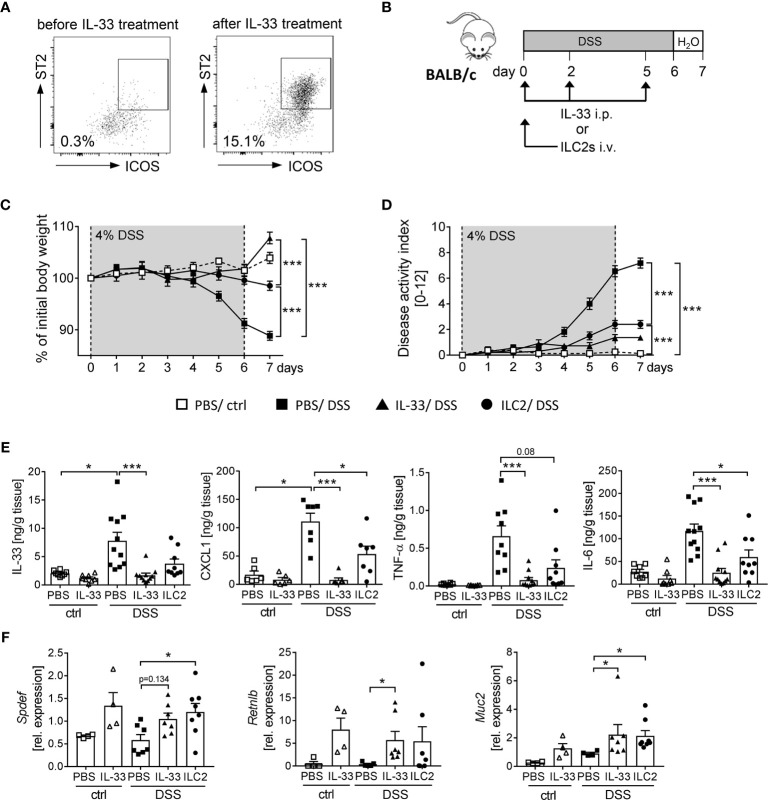
ILC2 transfer alleviates DSS-induced colitis. ILC2s were expanded *in vivo* in donor BALB/c mice by injecting IL-33 on day -7, -5, -3 and -1 prior to the transfer. **(A)** Representative dot plots show the expansion of ILC2s in the spleen upon IL-33 treatment gated on ST2^+^ICOS^+^ of viable Lin^-^ cells. **(B)** To induce intestinal inflammation, DSS was orally administered to BALB/c mice for 6 days, followed by one day of normal drinking water. Mice were either adoptively transferred i.v. with 2 x 10^5^ ILC2s on day 0 or treated with recombinant mIL-33 i.p. on day 0, 2 and 5 (n = 4-11 mice per group). **(C)** Body weight change and **(D)** disease activity index were monitored daily. **(E)** Secretion of IL-33, CXCL1/KC, TNF-α and IL-6 by colonic explants was quantified using Luminex technology. **(F)** Colonic mRNA expression of *Retnlb, Spdef* and *Muc2* were assessed by quantitative RT-PCR. All data are presented as mean ± SEM. Statistical analyses were performed using two-way ANOVA followed by Tukey’s multiple comparison test **(C, D)**, or one-way ANOVA followed by Dunn’s or Dunnett’s multiple comparison test **(E, F)**. *P < 0.05; ***P < 0.001.

We observed that the application of IL-33 strongly expands the frequency of ILC2s in WT and *RAG2* ^-/-^ mice (**Figures 4B**, **5G**). Thus, we aimed to determine the impact of ILC2s on intestinal inflammation in more detail. To obtain a sufficient number of ILC2 for transfer experiments, we expanded ILC2s in donor BALB/c mice with recombinant IL-33 (**Figure 6A**). At day 7, we FACS-purified splenic ILC2s from donor mice and adoptively transferred 2 x 10^5^ ILC2s into BALB/c mice before DSS treatment. To evaluate the impact of ILC2s during IL-33 treatment, we compared the ILC2 transfer with the injection of recombinant IL-33 (**Figure 6B**). Interestingly, ILC2-pre-transferred mice showed reduced intestinal inflammation as they lost less body weight and displayed a lower disease activity index in comparison to PBS-treated DSS mice. Nevertheless, the reduction in inflammation was less pronounced compared to IL-33-treated DSS-mice (**Figures 6C, D**). Consistently, the levels of IL-33, TNF-α, IL-6 and CXCL1 in the supernatant of colonic explants were decreased in both IL-33-treated and ILC2-pre-transferred DSS-mice (**Figure 6E**). As it was shown that IL-33 induces intestinal goblet cell differentiation indirectly through IL-13 production by ILC2s (21), we further assessed the impact of ILC2 transfer on goblet cells during DSS-induced colitis. Indeed, mice treated with recombinant IL-33 exhibited a restoration of crypt architecture in the histopathological analyses (**Figures 6D, E**), as well as elevated levels of *SAM (sterile alpha motif) pointed domain epithelia specific transcription factor* (*Spdef)* and *Resistin-like-beta* (*Retnlb*), transcription marker and secretory product of goblet cells, respectively. Furthermore, mice showed significant higher expression of the primary mucin *Muc2* upon IL-33 treatment, which indicates a reinforced goblet cell function (**Figure 6F**). Remarkably, we could show *in vivo* that ILC2-pre-transferred DSS mice exhibited a similar phenotype like IL-33-treated DSS mice with an increase of both goblet cell markers *Spdef* and *Retnlb* as well as an increase in the expression of *Muc2* in colonic tissues (**Figure 6F**). These results further endorse the protective function of IL-33 in severe, acute colitis by increasing the epithelial barrier function. In summary, we conclude that IL-33 not only exerted its effects *via* ILC2s, but we rather suggest a supportive function of Tregs to restrain DSS-induced intestinal inflammation.

## Discussion

The complex etiology of inflammatory bowel disease is still a subject of debate. However, an imbalance of cytokine production as well as T cell dysfunction are widely considered as key factors in the pathogenesis of mucosal inflammation (22). Recently, IL-33, as an IL-1 family cytokine and alarmin was ascribed a unique and essential role during colitis development (23, 24). Nonetheless, its dichotomous role in the intestinal tract led to conflicting results regarding a protective or pathogenic function in intestinal inflammation. On the one hand, IL-33 is considered to aggravate intestinal pathology, but on the other hand, it has been shown that IL-33 is able to attenuate colitis development (12, 14, 25, 26), a characteristic of IL-33 that was also observed for other disease patterns (27–30). Therefore, further understanding the role of the IL-33/ST2 signaling during DSS-induced colitis might reveal new therapeutic strategies to counteract intestinal inflammation.

The results presented here support the role of IL-33 as a potential therapeutic target during the acute phase of colitis, given the striking protection promoted by exogenous IL-33 treatment.

IL-33 is known to be constitutively expressed at high levels in the nucleus of epithelial cells (8) and its expression is further elevated in active lesions of IBD patients and mice with DSS-induced colitis (31–34). In accordance with these observations, we could show a strong increase of endogenous IL-33 expression in colonic biopsies of both IBD patients and DSS-treated mice, highlighting its role during intestinal inflammation and its potential as a biomarker in IBD. In contrast to results obtained by Lopetuso et al., who found a delayed recovery from DSS-induced colitis in *ST2^-/-^* mice but observed no differences in intestinal inflammation during the actual DSS challenge (35), we detected a strong enhancement of disease severity in *ST2^-/-^* mice compared to WT mice from day 4 onwards. These discrepancies might be attributed to different background of the mice or an altered DSS regimen. Well in line with increased intestinal inflammation in *ST2^-/-^* mice, our results further support a protective role for exogenous IL-33, as mice treated with recombinant IL-33 exhibited a tremendous alleviation of intestinal inflammation compared to PBS-treated DSS mice. More importantly, the strong increase of endogenous IL-33 was diminished when mice were treated exogenously with IL-33. These results emphasize the crucial difference between endogenous and exogenous IL-33 expression and may further explain the contradictory results concerning its colonic function in former studies. Consistent with this suggestion, He et al. could show a difference between full-length IL-33, which is present in the nucleus of epithelial cells and accumulates during intestinal inflammation, and mature IL-33, which is cleaved by different caspases and is found in the extracellular space. In detail, over-expression of full-length IL-33 by intestinal epithelial cells did not promote inflammation, but induced expression of genes that correlated with increased Th2 immune responses, such as *Gata3*, *Il4* and *Il13*. Therefore, it is proposed that the mature (cleaved) form rather than the nuclear (full-length) form of IL-33 mediates the transcriptional effects on intestinal epithelial cells (36).

Since IBD is characterized by uncontrolled activation of intestinal immune cells (37), we further assessed the impact of IL-33 on ST2^+^ immune cells during DSS-induced colitis. We could observe a pronounced expansion of Tregs and ILC2s in frequencies and absolute numbers in the colon of IL-33-treated DSS-mice and further suggested a need for these cells to trigger the protective effect orchestrated by IL-33 signaling. Well in line, previous studies emphasized a crucial role of IL-33 in innate immunity and inflammation by inducing ILC2 functions (38). In particular, studies investigating IL-33 in gut inflammation could show that recombinant IL-33 treatment resulted in an improved outcome of experimental intestinal inflammation in mice through the activation of ILC2s in an AREG-dependent manner or through the induction of CD103^+^ DCs to promote the development of Tregs *via* IL-2 secretion (25, 39, 40). Tregs are known to play a crucial role in the pathogenesis of colitis, as their frequencies are altered in IBD patients (41, 42) and as they exert various immunosuppressive mechanisms like downregulating Th17 responsiveness *via* TGF-β (43). In our study, we could demonstrate, by using DEREG mice, that the specific depletion of Foxp3^+^ Tregs partially abrogated the positive effects of IL-33 administration in DSS colitis. This provides evidence that Foxp3^+^ Tregs are involved in IL-33 mediated tissue protection. However, our experiments in *RAG2^-/-^* mice and the fact that a part of the IL-33-protective effect was still present after Treg depletion further indicate a non-exclusive role for Tregs in conveying mucosal protection. Similarly, Duan et al. could show that depletion of Tregs *via* anti-CD25 application significantly abrogated the impact of IL-33 on reducing the development of colitis using the TNBS-induced colitis model (40). While Duan et al. claim that IL-33 triggers a Th1-to-Th2/Treg switch, which mediates improved colon pathology, we propose that IL-33-induced alleviation of DSS colitis is only partially dependent on Tregs. Furthermore, we could show that DSS dependent pro-inflammatory cytokine production was still reduced in Treg-depleted mice comparable to Treg-sufficient mice after IL-33 treatment, whereas Th2-associated cytokines were no longer induced after Treg depletion. Therefore, our data might indicate that Tregs are supportive to other immune cells to produce IL-13 and IL-5. As IL-13, secreted by ILC2s, promotes regeneration of the intestinal epithelium (44), the reduction in IL-13 expression in turn could be one reason for a less favorable outcome of IL-33 treatment in Treg-depleted mice.

Hence, we further focused on cells from the innate immune system as we could also observe a remarkably increase of ILC2s in DSS-mice after IL-33 application. ILC2s are considered to contribute to the maintenance of epithelial integrity in the gut and have been shown to increase upon IL-33 stimulation (45). Similar to the diverse function of IL-33, ILC2s may reflect different roles regarding the regulation of pro-inflammatory and tissue-protective responses at mucosal barrier sites. While some studies found positive correlations of ILC2 frequencies and disease severity in different colitis models (13, 46), Monticelli et al. identified a host-protective IL-33-driven mechanism, involving the promotion of ILC2s and consequently the expression of growth factors essential for tissue protection and the restoration of intestinal homeostasis (25). Likewise, we demonstrated that wild-type mice pre-transferred with activated ILC2s displayed less signs of inflammation, indicated by an improved body weight, lower disease activity index and reduction of pro-inflammatory cytokine secretion. Mechanistically, we propose that IL-33-activated ILC2s produce Th2-associated cytokines, like IL-13, thereby promoting goblet cell differentiation and subsequent epithelial barrier integrity. In this context, IL-13 expression might be of central importance in intestinal inflammation. In response to IL-33, ILC2s are the major source of IL-13 among innate immune cells (47) and are able to promote goblet cell expansion (21). During IBD, IL-13 impairs the production of IL-1β, TNF-α and IL-6 (48). Still, IL-13 secretion is not the only mechanism of ILC2s to protect mice from intestinal inflammation. A study conducted by You et al. could show that the transfer of activated ILC2s was able to alleviate DSS-induced acute colitis in *RAG1^-/-^* mice by promoting M2 macrophage polarization (49) and Monticelli et al. emphasize amphiregulin production by ILC2s to enhance mucin production and reduce intestinal inflammation in DSS treated *AREG^-/-^* mice (25). Importantly, in contrast to the aforementioned studies, we could demonstrate that also DSS treated wild-type mice benefited from the transfer of IL-33 expanded ILC2s and that these mice displayed a phenotype comparable to IL-33 treated DSS mice. Nevertheless, the alleviating impact of ILC2 transfer was not as strong as IL-33 treatment, further supporting a collaborative role for other factors or immune cells, to restrain acute colitis.

With its diverse functions, IL-33 could be the linking mediator between different ST2^+^ immune cells. Indeed, it has already been demonstrated that IL-33 is able to act indirectly to enhance Treg numbers and function through the promotion of ILC2s (50). Nascimento et al. could show that IL-33-driven ILC2 activation promoted the polarization of M2 macrophages and consequently the expansion of Tregs through the secretion of IL-10. Both indicating not only a crosslink between ILC2s and Tregs, but rather emphasize a promoting capacity of ILC2s to induce Tregs in an indirect manner. Nevertheless, we cannot rule out that other immune cells than Tregs interact with ILC2s to promote their protective function during intestinal inflammation. This assumption is supported by our investigations in *RAG2^-/-^* mice that lack mature T and B cells. Here, IL-33 treatment potently ameliorated DSS-induced inflammation and expanded colonic ILC2s, as well as IL-5 and IL-13 secretion. Possibly, ILC2s also cooperate with eosinophils due to their constitutive expression of IL-5 that was demonstrated to regulate both eosinophil homeostasis as well as their tissue accumulation and infiltration into the colon (51, 52).

Overall, our results suggest that ILC2s facilitate IL-33-mediated tissue protection in DSS colitis, while Tregs seem to play an ILC2-supporting role. This could be due to the fact that ILC2s constitutively express the receptor ST2^+^ and are therefore able to act immediately upon IL-33 treatment, whereas only a subpopulation of Tregs display ST2 expression. However, given the divers function of each cell type upon IL-33 treatment during intestinal inflammation, additional experiments are necessary to accurately identify the mechanisms of ILC2-driven mucosal protection.

In summary, in this study we highlight the potential of exogenous IL-33 application during acute colitis and its essential role for promoting ILC2 activation to restrain intestinal inflammation.

## Data Availability Statement

The raw data supporting the conclusions of this article will be made available by the authors, without undue reservation.

## Ethics Statement

The studies involving human participants were reviewed and approved by Faculty of the University of Duisburg-Essen. The patients/participants provided their written informed consent to participate in this study. The animal study was reviewed and approved by Landesamt für Natur-, Umwelt- und Verbraucherschutz (LANUV, North-Rhine-Westphalia, Germany).

## Author Contributions

NN performed the experiments, analyzed the data and wrote the manuscript. AW supervised the study and wrote the manuscript. EP designed and supervised the study, and wrote the manuscript. JL provided human samples. RP performed all histological analyses. VP, AA, and WH revised the manuscript. All authors contributed to the article and approved the submitted version.

## Funding

This work was supported by the German Research Foundation (DFG) research grant GRK 1949 to AW and PA 2792/2-1 to EP. We acknowledge support by the Open Access Publication Fund of the University of Duisburg-Essen.

## Conflict of Interest

The authors declare that the research was conducted in the absence of any commercial or financial relationships that could be construed as a potential conflict of interest.
