# Supplementary material for: IL-33 Drives Expansion of Type 2 Innate Lymphoid Cells and Regulatory T Cells and Protects Mice From Severe, Acute Colitis
Source: Front Immunol. 2021 Jul 15;12:669787. doi: 10.3389/fimmu.2021.669787 (PMC8320374; doi:10.3389/fimmu.2021.669787)
Supplement: Supplementary file 1 [file DataSheet_1.pdf]

Supplementary Figure 1

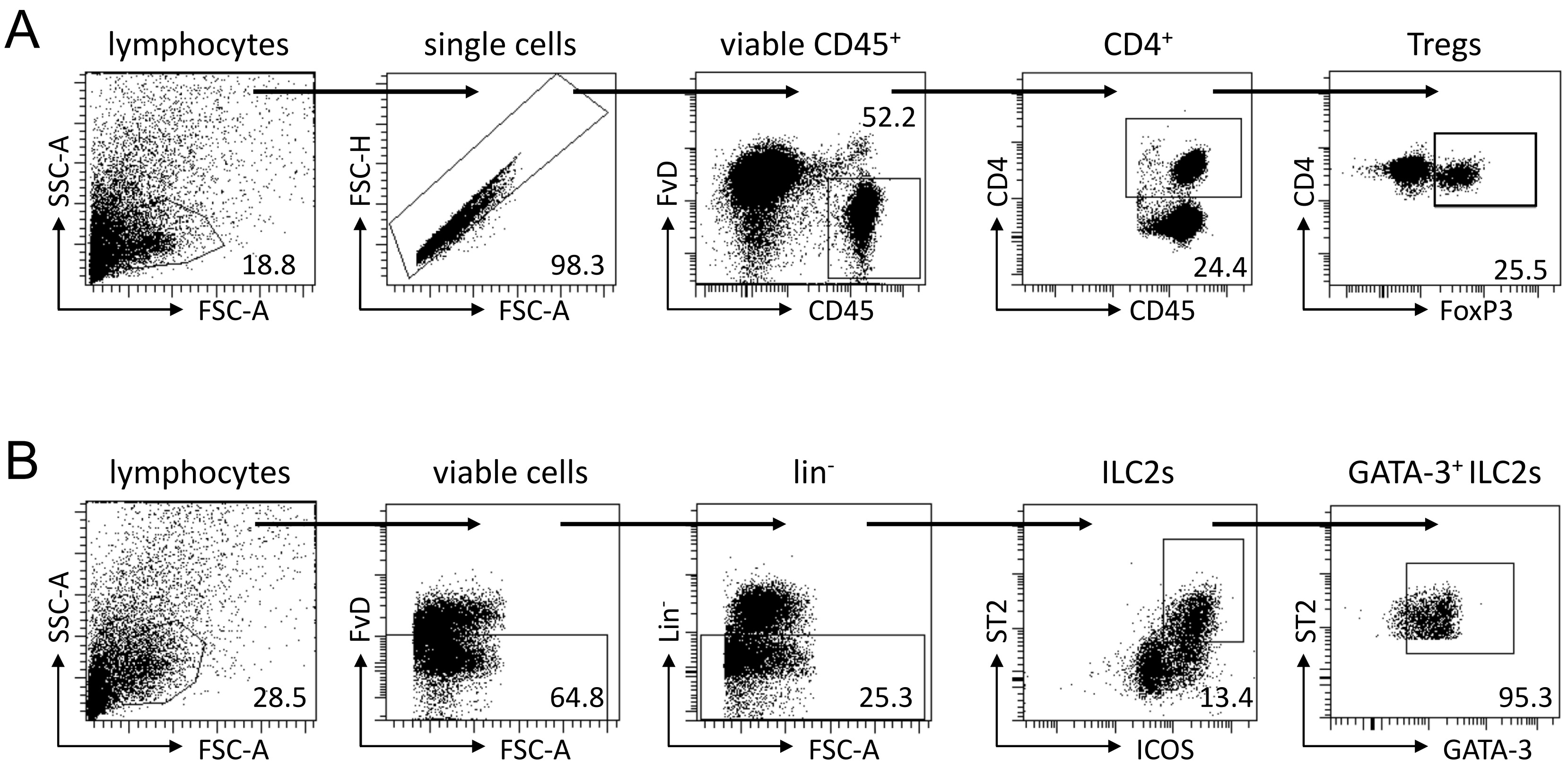

Supplementary Fig. 1. Gating strategy of regulatory T cells and type 2 innate lymphoid cells. To induce intestinal inflammation, BALB/c mice were given 4% of DSS for six consecutive days, followed by one day of normal drinking water. Mice were injected i.p. either with PBS or with IL-33 on day 0, 2 and 5. Immune cells were isolated from the colonic lamina propria on day 7 and analyzed by flow cytometry to distinguish (A) $CD4^+Foxp3^+$ regulatory T cells (Tregs), or (B) $lineage^-ICOS^+ST2^+$ type 2 innate lymphoid cells (ILC2s). Representative dot plots show the gating strategy of Tregs and ILC2s, and the expression of GATA-3 among ILC2s from the colon of an IL-33 treated DSS mouse.
